# Supplementary material for: Comparative In Vitro Toxicology of Novel Cytoprotective Short-Chain Naphthoquinones
Source: Pharmaceuticals (Basel). 2020 Aug 7;13(8):184. doi: 10.3390/ph13080184 (PMC7463972; doi:10.3390/ph13080184)
Supplement: Supplementary file 1 [file pharmaceuticals-13-00184-s001.pdf]

Supplementary Materials

Comparative In Vitro Toxicology of Novel Cytoprotective Short-Chain Naphthoquinones

Zikai Feng [1,2,*], Mohammed Sedeeq [1], Abraham Daniel [1], Monika Corban [1], Krystel L. Woolley [2], Ryan Condie [2], Iman Azimi [1], Jason A. Smith [2] and Nuri Gueven [1,*]

[1] School of Pharmacy and Pharmacology, University of Tasmania, Hobart, TAS 7005, Australia; mohammed.sedeeq@utas.edu.au (M.S.); abraham.daniel@utas.edu.au (A.D.); monika.corban@utas.edu.au (M.C.); iman.azimi@utas.edu.au (I.A.)

[2] School of Natural Sciences, University of Tasmania, Hobart, TAS 7005, Australia; krystel.woolley@utas.edu.au (K.L.W.); ryan.condie@utas.edu.au (R.C.); jason.smith@utas.edu.au (J.A.S.)

* Correspondence: zikai.feng@utas.edu.au (Z.F.); nuri.guven@utas.edu.au (N.G.)

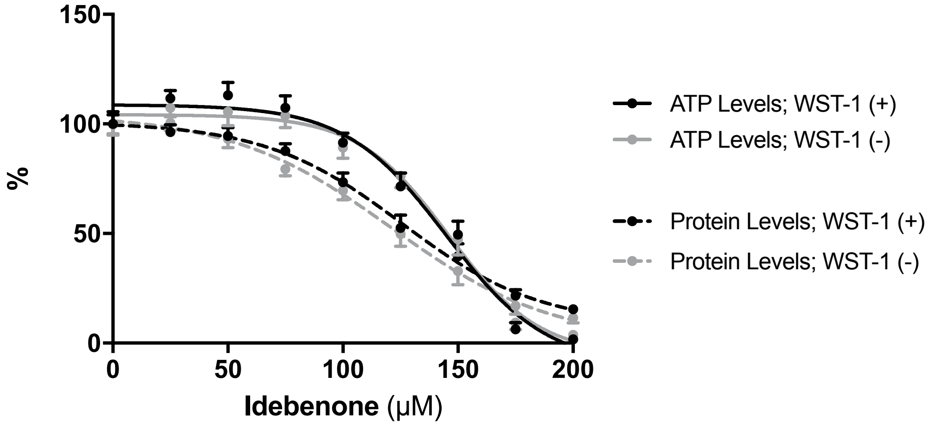

Figure S1. Validation of multiplex detection of NAD(P)H, ATP and Protein Levels. Cells were exposed to idebenone (0–200 µM) for 24 h before WST-1 absorption, ATP levels and protein contents were quantified. Data represents mean ± standard error of mean (SEM) from 3 independent experiments with 4 parallel wells per experiment. Two-way ANOVA was performed, no statistically significant differences were observed over a range of concentrations with (black lines) or without (gray lines) NAD(P)H measurement prior to the quantitation of ATP (solid lines) and protein (dotted lines) content from cell lysates.

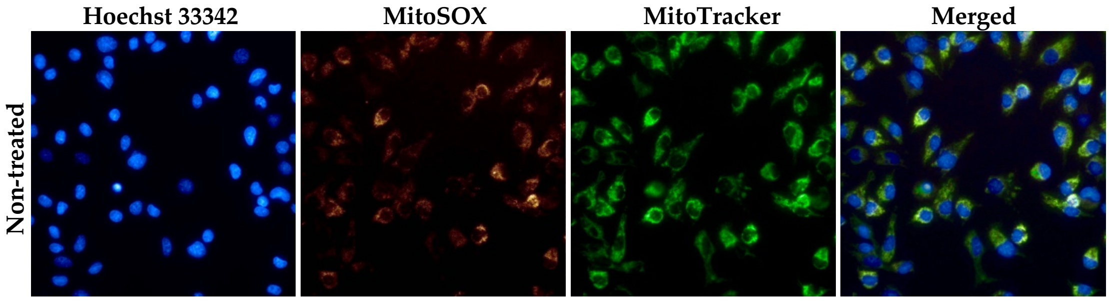

Figure S2. Exemplary images of MitoSOX localization in HepG2 cells used to measure mitochondrial superoxide production. Cells were seeded on poly-*L*-lysine pre-coated plates, left to adhere, loaded with MitoSOX Red and Hoechst 33342 (methods detailed in main text 4.6) and MitoTracker Green (0.1 µM in HBSS + 1% BSA, 30 µL/well; M7514, Thermo Fisher Scientific, Scoresby, VIC, Australia) to confirm mitochondrial localization, and imaged using an IN Cell 2200 analyzer (60 × magnification, GE Healthcare, Rydalmere, NSW, Australia).

Table S1. Effect of test compounds on WST-1 absorption.

μM	0	25	50	75	100	125	150	175	200
Idebenone	100.0 ± 2.1	106.9 ± 3.3	122.4 ± 4.8	136.9 ± 6.9	124.6 ± 7.5	114.0 ± 8.7	75.9 ± 8.0	40.8 ± 4.9	30.5 ± 1.7
**1**	100.0 ± 2.8	119.8 ± 3.6	86.9 ± 3.4	34.8 ± 5.7	21.7 ± 1.2	19.6 ± 0.6	19.8 ± 0.7	19.7 ± 0.5	19.0 ± 0.5
**2**	100.0 ± 3.2	120.8 ± 7.2	48.7 ± 2.8	26.9 ± 1.9	19.7 ± 0.8	18.3 ± 0.7	18.3 ± 0.7	18.8 ± 0.8	18.9 ± 0.9
**3**	100.0 ± 4.5	87.2 ± 2.9	81.1 ± 4.9	66.8 ± 3.9	55.2 ± 2.6	46.6 ± 1.2	43.4 ± 0.8	41.6 ± 0.8	41.2 ± 0.9
**4**	100.0 ± 5.0	111.8 ± 6.6	107.0 ± 3.9	104.7 ± 2.7	97.6 ± 3.3	95.9 ± 4.2	92.3 ± 4.5	88.0 ± 2.8	84.7 ± 2.6
**5**	100.0 ± 2.8	111.9 ± 4.6	126.7 ± 4.7	112.4 ± 2.2	112.9 ± 3.3	105.6 ± 2.7	93.3 ± 4.3	77.9 ± 3.0	67.9 ± 3.0
**6**	100.0 ± 1.9	116.0 ± 2.8	104.5 ± 2.2	53.9 ± 2.4	34.2 ± 2.5	29.1 ± 1.5	27.3 ± 1.4	27.2 ± 1.7	26.1 ± 1.3
**7**	100.0 ± 3.4	121.3 ± 5.6	100.1 ± 4.8	85.2 ± 5.7	57.9 ± 1.2	54.4 ± 2.0	52.9 ± 1.0	49.5 ± 1.2	47.3 ± 0.5
**8**	100.0 ± 1.8	83.2 ± 2.6	78.7 ± 4.5	70.9 ± 3.0	50.3 ± 1.6	42.4 ± 1.3	42.4 ± 1.1	40.3 ± 1.0	40.1 ± 0.8
**9**	100.0 ± 3.9	115.4 ± 3.4	80.5 ± 3.9	39.9 ± 2.3	32.8 ± 2.7	30.2 ± 2.9	29.4 ± 2.4	31.0 ± 2.2	30.3 ± 2.0
**10**	100.0 ± 2.9	114.4 ± 3.1	67.6 ± 2.9	49.5 ± 4.3	38.4 ± 1.9	34.3 ± 1.2	35.2 ± 0.9	34.6 ± 1.3	34.4 ± 1.1
**11**	100.0 ± 1.6	89.0 ± 2.3	84.7 ± 3.8	78.2 ± 3.2	65.3 ± 2.7	48.8 ± 2.2	42.2 ± 2.2	40.3 ± 2.4	38.5 ± 2.9

Data was standardized and expressed as mean ± SEM (%) of 3 independent experiments with 6 parallel wells per experiment.

Table S2. Effect of test compounds on ATP levels.

μM	0	25	50	75	100	125	150	175	200
Idebenone	100.0 ± 5.4	111.1 ± 3.4	112.4 ± 5.6	106.8 ± 5.1	91.5 ± 4.2	72.2 ± 6.0	50.9 ± 6.0	9.6 ± 3.1	5.3 ± 0.6
**1**	100.0 ± 1.9	109.9 ± 4.6	61.4 ± 4.7	21.4 ± 3.9	4.8 ± 1.6	0.7 ± 0.2	0.6 ± 0.2	0.2 ± 0.1	2.3 ± 0.6
**2**	100.0 ± 2.3	96.5 ± 4.5	51.4 ± 5.7	22.5 ± 4.0	7.3 ± 1.7	1.7 ± 0.6	1.7 ± 1.3	0.1 ± 0.1	0.8 ± 0.4
**3**	100.0 ± 5.4	100.1 ± 4.3	82.6 ± 4.8	71.2 ± 3.7	49.9 ± 2.0	39.8 ± 2.3	30.3 ± 2.9	23.2 ± 3.4	23.7 ± 4.0
**4**	100.0 ± 4.3	107.0 ± 5.9	92.8 ± 5.0	98.3 ± 4.4	88.1 ± 6.1	83.3 ± 5.7	90.4 ± 5.0	92.0 ± 5.3	75.6 ± 3.7
**5**	100.0 ± 2.5	108.2 ± 3.2	106.9 ± 3.9	106.5 ± 3.7	105.5 ± 3.9	92.8 ± 4.3	84.7 ± 3.8	70.8 ± 3.0	60.9 ± 3.9
**6**	100.0 ± 2.9	103.1 ± 4.9	78.2 ± 2.8	41.3 ± 3.5	14.0 ± 3.0	5.1 ± 0.5	4.0 ± 0.6	4.5 ± 0.9	5.2 ± 0.6
**7**	100.0 ± 2.4	112.5 ± 3.8	93.5 ± 4.1	66.4 ± 3.6	51.2 ± 2.1	41.1 ± 1.9	37.7 ± 1.0	33.3 ± 1.5	25.7 ± 1.3
**8**	100.0 ± 2.2	90.8 ± 1.9	87.5 ± 4.2	78.0 ± 4.9	45.8 ± 3.8	26.7 ± 2.0	22.9 ± 1.9	21.4 ± 1.0	19.3 ± 1.7
**9**	100.0 ± 6.2	100.5 ± 6.8	57.3 ± 4.0	22.1 ± 2.5	15.7 ± 3.2	9.0 ± 2.1	6.8 ± 1.5	8.2 ± 1.0	6.8 ± 0.6
**10**	100.0 ± 3.7	93.1 ± 3.2	57.6 ± 4.1	27.0 ± 4.0	6.1 ± 1.5	3.1 ± 1.0	3.0 ± 0.7	4.2 ± 0.8	6.8 ± 1.2
**11**	100.0 ± 2.8	97.6 ± 3.7	95.0 ± 3.0	80.5 ± 4.0	58.5 ± 2.0	37.9 ± 2.9	28.8 ± 2.0	26.2 ± 1.8	19.5 ± 1.0

Data was standardized and expressed as mean ± SEM (%) of 3 independent experiments with 6 parallel wells per experiment.

Table S3. Effect of test compounds on protein levels.

μM	0	25	50	75	100	125	150	175	200
Idebenone	100.0 ± 4.2	96.3 ± 3.3	94.5 ± 3.9	87.7 ± 3.3	73.4 ± 4.2	52.6 ± 5.8	39.6 ± 5.6	21.7 ± 2.7	15.4 ± 1.3
**1**	100.0 ± 1.9	98.3 ± 1.5	74.5 ± 1.4	44.4 ± 3.3	27.5 ± 1.8	23.3 ± 0.9	25.9 ± 2.1	20.3 ± 0.5	24.1 ± 1.1
**2**	100.0 ± 1.7	93.2 ± 1.9	66.7 ± 3.4	46.7 ± 3.4	36.6 ± 2.8	28.8 ± 1.0	33.8 ± 3.0	28.1 ± 1.4	33.7 ± 2.0
**3**	100.0 ± 4.2	100.3 ± 2.7	79.5 ± 3.2	58.8 ± 2.4	49.8 ± 1.9	44.5 ± 2.5	41.8 ± 2.1	26.0 ± 2.1	32.4 ± 2.7
**4**	100.0 ± 5.9	100.8 ± 5.1	87.2 ± 5.6	92.8 ± 4.5	81.2 ± 4.7	74.0 ± 3.6	76.7 ± 2.9	78.0 ± 3.6	61.8 ± 2.1
**5**	100.0 ± 2.2	100.8 ± 3.6	91.7 ± 3.5	93.2 ± 4.2	79.2 ± 2.3	79.2 ± 3.0	72.9 ± 3.3	70.0 ± 3.2	65.7 ± 4.2
**6**	100.0 ± 2.2	96.0 ± 3.2	79.4 ± 4.5	46.2 ± 3.4	26.8 ± 3.0	18.6 ± 2.3	17.5 ± 2.3	19.4 ± 2.0	15.2 ± 2.0
**7**	100.0 ± 3.0	88.6 ± 2.3	73.3 ± 3.2	56.3 ± 3.1	39.9 ± 2.4	34.8 ± 2.6	37.7 ± 3.8	37.6 ± 2.8	28.1 ± 1.8
**8**	100.0 ± 6.9	100.2 ± 6.8	96.8 ± 5.6	73.4 ± 5.6	49.2 ± 3.4	33.8 ± 3.2	35.5 ± 3.2	40.1 ± 4.1	29.6 ± 2.7
**9**	100.0 ± 4.9	87.6 ± 3.9	68.5 ± 4.0	38.3 ± 4.3	23.5 ± 3.7	18.9 ± 2.9	18.7 ± 2.9	19.2 ± 2.3	18.5 ± 2.1
**10**	100.0 ± 3.8	87.6 ± 5.9	75.0 ± 5.1	45.8 ± 5.4	31.7 ± 2.4	26.5 ± 2.1	27.8 ± 1.4	26.7 ± 2.0	23.3 ± 1.7
**11**	100.0 ± 3.6	96.1 ± 5.3	89.7 ± 4.2	68.4 ± 4.0	61.1 ± 2.4	40.0 ± 3.0	29.8 ± 2.0	33.5 ± 2.1	30.8 ± 4.4

Data was standardized and expressed as mean ± SEM (%) of 3 independent experiments with 6 parallel wells per experiment.

Table S4. Effect of test compounds on propidium iodide (PI) incorporation.

μM	0	25	50	75	100	125	150	175	200
Idebenone	1.0 ± 0.0	1.0 ± 0.0	1.0 ± 0.1	1.4 ± 0.1	1.7 ± 0.2	3.8 ± 0.1	3.1 ± 0.3	3.6 ± 0.1	3.7 ± 0.1
**1**	1.0 ± 0.1	1.2 ± 0.1	1.8 ± 0.1	2.7 ± 0.3	4.0 ± 0.1	4.2 ± 0.3	4.0 ± 0.2	4.3 ± 0.2	3.6 ± 0.1
**2**	1.0 ± 0.1	1.2 ± 0.1	2.1 ± 0.3	3.1 ± 0.3	3.7 ± 0.3	4.6 ± 0.7	4.6 ± 0.3	3.9 ± 0.3	4.1 ± 0.3
**3**	1.0 ± 0.1	1.0 ± 0.1	1.2 ± 0.2	2.4 ± 0.3	3.4 ± 0.1	3.2 ± 1.2	4.5 ± 0.4	4.4 ± 0.3	4.3 ± 0.4
**4**	1.0 ± 0.1	1.0 ± 0.1	1.0 ± 0.1	0.8 ± 0.1	1.0 ± 0.1	1.1 ± 0.2	1.4 ± 0.1	1.5 ± 0.1	1.6 ± 0.3
**5**	1.0 ± 0.1	1.1 ± 0.2	1.0 ± 0.0	1.1 ± 0.0	1.1 ± 0.1	1.5 ± 0.0	2.0 ± 0.2	3.7 ± 0.3	3.8 ± 0.3
**6**	1.0 ± 0.1	1.2 ± 0.1	1.6 ± 0.2	2.6 ± 0.2	3.7 ± 0.2	3.2 ± 0.1	4.2 ± 0.3	4.9 ± 0.4	4.6 ± 0.4
**7**	1.0 ± 0.1	1.1 ± 0.1	1.4 ± 0.2	1.7 ± 0.2	2.9 ± 0.2	3.9 ± 0.2	4.5 ± 0.4	3.4 ± 0.2	3.8 ± 0.5
**8**	1.0 ± 0.1	0.9 ± 0.1	1.0 ± 0.1	1.2 ± 0.1	1.4 ± 0.1	3.8 ± 0.2	3.8 ± 0.4	3.1 ± 0.4	3.1 ± 0.1
**9**	1.0 ± 0.1	0.9 ± 0.1	1.4 ± 0.1	3.0 ± 0.2	3.8 ± 0.2	5.0 ± 0.2	4.7 ± 0.5	4.6 ± 0.5	3.4 ± 0.2
**10**	1.0 ± 0.1	0.9 ± 0.1	1.0 ± 0.1	2.5 ± 0.2	3.1 ± 0.2	4.2 ± 0.3	4.3 ± 0.3	3.8 ± 0.5	4.1 ± 0.5
**11**	1.0 ± 0.1	0.7 ± 0.1	1.2 ± 0.2	0.8 ± 0.1	0.8 ± 0.1	1.7 ± 0.1	3.0 ± 0.3	3.7 ± 0.2	3.6 ± 0.5

Data was standardized and expressed as mean ± SEM (fold) of 3 independent experiments with 4 parallel wells per experiment.

Table S5. Effect of test compounds on necrotic-cell protease activity.

μM	0	25	50	75	100	125	150	175	200
Idebenone	1.0 ± 0.0	0.9 ± 0.1	1.0 ± 0.1	1.0 ± 0.1	1.7 ± 0.3	1.7 ± 0.6	1.1 ± 0.1	0.8 ± 0.1	0.2 ± 0.2
**1**	1.0 ± 0.0	1.5 ± 0.1	1.7 ± 0.1	1.8 ± 0.2	1.4 ± 0.2	1.4 ± 0.4	1.5 ± 0.3	1.4 ± 0.3	1.3 ± 0.4
**2**	1.0 ± 0.0	1.5 ± 0.1	1.6 ± 0.2	1.7 ± 0.2	1.7 ± 0.2	1.9 ± 0.6	1.6 ± 0.5	1.0 ± 0.2	0.8 ± 0.1
**3**	1.0 ± 0.0	1.7 ± 0.2	2.0 ± 0.3	2.0 ± 0.3	1.6 ± 0.2	1.7 ± 0.4	1.3 ± 0.3	0.7 ± 0.1	0.7 ± 0.2
**4**	1.0 ± 0.0	0.9 ± 0.1	0.8 ± 0.1	1.3 ± 0.2	0.9 ± 0.1	1.3 ± 0.3	1.0 ± 0.1	0.8 ± 0.2	0.9 ± 0.2
**5**	1.0 ± 0.0	0.6 ± 0.1	1.0 ± 0.1	1.5 ± 0.2	1.9 ± 0.5	1.6 ± 0.2	1.2 ± 0.2	1.2 ± 0.3	1.3 ± 0.3
**6**	1.0 ± 0.0	1.4 ± 0.2	1.4 ± 0.1	1.5 ± 0.2	1.2 ± 0.3	2.0 ± 0.4	2.1 ± 0.6	1.8 ± 0.5	1.8 ± 0.6
**7**	1.0 ± 0.0	1.3 ± 0.1	1.6 ± 0.2	1.5 ± 0.2	1.8 ± 0.4	2.9 ± 0.8	3.0 ± 0.7	3.0 ± 1.8	3.1 ± 0.6
**8**	1.0 ± 0.0	1.0 ± 0.1	1.3 ± 0.2	1.4 ± 0.1	1.8 ± 0.3	2.5 ± 0.4	2.6 ± 0.4	2.7 ± 0.5	3.0 ± 0.7
**9**	1.0 ± 0.0	1.5 ± 0.1	1.5 ± 0.2	1.5 ± 0.2	1.9 ± 0.4	2.7 ± 0.5	2.7 ± 0.5	2.9 ± 0.9	3.1 ± 0.7
**10**	1.0 ± 0.0	1.1 ± 0.1	1.3 ± 0.1	1.3 ± 0.2	1.4 ± 0.3	1.9 ± 0.3	2.0 ± 0.3	1.8 ± 0.2	2.0 ± 0.5
**11**	1.0 ± 0.0	1.1 ± 0.2	1.2 ± 0.1	1.2 ± 0.2	1.7 ± 0.3	2.4 ± 0.2	2.5 ± 0.4	2.3 ± 0.5	2.5 ± 0.8

Data was standardized and expressed as mean ± SEM (fold) of 3 independent experiments with 4 parallel wells per experiment.

Table S6. Effect of test compounds on viable-cell protease activity.

μM	0	25	50	75	100	125	150	175	200
Idebenone	100.0 ± 1.5	106.0 ± 7.2	84.7 ± 8.8	54.8 ± 11.6	41.9 ± 7.5	27.0 ± 6.1	21.5 ± 2.8	21.5 ± 4.1	25.9 ± 6.7
**1**	100.0 ± 1.5	92.6 ± 8.9	64.7 ± 9.5	52.8 ± 7.7	36.8 ± 6.0	32.5 ± 7.8	32.9 ± 5.2	32.0 ± 5.5	30.5 ± 5.1
**2**	100.0 ± 1.5	80.8 ± 8.6	59.2 ± 7.7	42.2 ± 6.2	30.7 ± 5.1	30.9 ± 6.5	27.9 ± 5.3	23.2 ± 2.3	24.8 ± 4.7
**3**	100.0 ± 1.5	91.7 ± 6.8	65.9 ± 10.1	53.1 ± 8.8	37.6 ± 6.3	33.7 ± 7.0	27.8 ± 5.2	24.9 ± 3.4	24.1 ± 3.0
**4**	100.0 ± 1.5	109.9 ± 6.9	105.0 ± 8.0	110.9 ± 7.4	95.2 ± 8.1	89.3 ± 13.9	80.3 ± 9.6	71.6 ± 5.5	63.9 ± 9.0
**5**	100.0 ± 1.5	100.0 ± 6.1	96.6 ± 9.4	98.6 ± 9.0	93.6 ± 10.6	73.6 ± 12.6	64.0 ± 9.8	60.0 ± 5.4	43.5 ± 5.4
**6**	100.0 ± 1.5	85.4 ± 7.1	55.9 ± 8.2	40.1 ± 5.7	26.8 ± 4.2	32.3 ± 4.9	28.1 ± 3.2	25.8 ± 3.1	24.6 ± 2.3
**7**	100.0 ± 1.5	78.3 ± 5.7	56.8 ± 9.6	45.9 ± 7.2	43.8 ± 8.7	35.5 ± 8.7	31.7 ± 5.0	29.5 ± 3.4	27.6 ± 3.9
**8**	100.0 ± 1.5	102.4 ± 4.1	78.0 ± 6.0	66.5 ± 8.7	62.9 ± 8.2	57.8 ± 9.1	54.7 ± 8.6	47.0 ± 5.2	46.5 ± 4.8
**9**	100.0 ± 1.5	83.2 ± 5.5	56.8 ± 6.8	43.3 ± 8.0	38.8 ± 6.8	38.6 ± 5.8	32.9 ± 2.8	29.5 ± 2.6	28.5 ± 2.2
**10**	100.0 ± 1.5	93.7 ± 8.2	63.2 ± 9.9	42.1 ± 7.3	32.8 ± 5.8	30.4 ± 5.3	29.5 ± 3.5	27.1 ± 2.5	25.4 ± 1.7
**11**	100.0 ± 1.5	104.8 ± 5.6	95.4 ± 6.5	93.2 ± 6.9	84.7 ± 10.1	89.0 ± 10.9	81.8 ± 8.2	77.1 ± 4.3	66.2 ± 4.4

Data was standardized and expressed as mean ± SEM (%) of 3 independent experiments with 4 parallel wells per experiment.

Table S7. Effect of test compounds on mitochondrial superoxide production.

μM	0	25	50	75	100	125	150	175	200
Antimycin A	1.0 ± 0.1	3.9 ± 0.7	4.8 ± 0.8	5.8 ± 1.2	6.3 ± 1.2	7.5 ± 1.5	7.7 ± 1.5	8.6 ± 1.7	9.0 ± 1.9
Idebenone	1.0 ± 0.1	1.3 ± 0.2	0.9 ± 0.0	0.9 ± 0.1	0.9 ± 0.0	0.9 ± 0.1	0.9 ± 0.0	0.9 ± 0.1	0.9 ± 0.0
**1**	1.0 ± 0.1	1.0 ± 0.1	1.0 ± 0.1	1.0 ± 0.1	1.0 ± 0.1	1.1 ± 0.1	1.1 ± 0.1	1.2 ± 0.1	1.2 ± 0.1
**2**	1.0 ± 0.1	1.1 ± 0.1	1.0 ± 0.1	1.0 ± 0.1	1.0 ± 0.1	1.0 ± 0.1	1.0 ± 0.1	1.0 ± 0.0	1.0 ± 0.1
**3**	1.0 ± 0.1	1.0 ± 0.1	1.0 ± 0.1	1.0 ± 0.0	1.0 ± 0.1	1.0 ± 0.1	1.0 ± 0.1	1.0 ± 0.1	1.0 ± 0.1
**4**	1.0 ± 0.1	1.4 ± 0.2	1.1 ± 0.1	1.0 ± 0.0	1.1 ± 0.1	1.1 ± 0.1	1.1 ± 0.1	1.1 ± 0.1	1.1 ± 0.1
**5**	1.0 ± 0.1	1.2 ± 0.1	1.2 ± 0.1	1.2 ± 0.1	1.2 ± 0.1	1.3 ± 0.1	1.4 ± 0.1	1.4 ± 0.1	1.5 ± 0.1
**6**	1.0 ± 0.1	1.1 ± 0.1	1.1 ± 0.0	1.0 ± 0.1	1.0 ± 0.1	1.0 ± 0.1	1.0 ± 0.1	1.0 ± 0.1	1.0 ± 0.1
**7**	1.0 ± 0.1	1.0 ± 0.1	1.0 ± 0.1	1.0 ± 0.1	1.0 ± 0.1	1.0 ± 0.1	1.0 ± 0.1	1.0 ± 0.1	1.0 ± 0.1
**8**	1.0 ± 0.1	1.3 ± 0.1	1.3 ± 0.1	1.4 ± 0.1	1.5 ± 0.2	1.6 ± 0.2	1.8 ± 0.2	1.9 ± 0.3	2.0 ± 0.3
**9**	1.0 ± 0.1	1.0 ± 0.1	1.1 ± 0.2	1.0 ± 0.1	1.0 ± 0.1	1.0 ± 0.1	1.0 ± 0.1	1.0 ± 0.1	1.0 ± 0.1
**10**	1.0 ± 0.1	1.1 ± 0.1	1.0 ± 0.1	1.1 ± 0.1	1.1 ± 0.1	1.1 ± 0.1	1.1 ± 0.1	1.2 ± 0.1	1.1 ± 0.1
**11**	1.0 ± 0.1	1.2 ± 0.1	1.3 ± 0.1	1.4 ± 0.1	1.6 ± 0.2	1.8 ± 0.2	2.0 ± 0.2	2.2 ± 0.3	2.2 ± 0.3

Data was standardized and expressed as mean ± SEM (fold) of 3 independent experiments with 8 parallel wells per experiment.

Table S8. Effect of test compounds on colony formation.

μM	0	5	10	15	20	40	60	80	100
Idebenone	100.0 ± 2.7	95.3 ± 4.3	84.6 ± 4.5	70.3 ± 4.2	68.3 ± 6.0	18.4 ± 3.1	0.7 ± 0.3	0.0 ± 0.0	0.0 ± 0.0
**1**	100.0 ± 4.2	58.5 ± 5.2	14.8 ± 6.2	11.3 ± 4.9	2.5 ± 1.2	0.0 ± 0.0	0.0 ± 0.0	0.0 ± 0.0	0.0 ± 0.0
**2**	100.0 ± 4.2	31.7 ± 2.7	0.1 ± 0.1	0.0 ± 0.0	0.0 ± 0.0	0.0 ± 0.0	0.0 ± 0.0	0.0 ± 0.0	0.0 ± 0.0
**3**	100.0 ± 2.6	59.8 ± 5.4	33.1 ± 6.4	10.0 ± 2.6	4.8 ± 1.0	0.1 ± 0.1	0.0 ± 0.0	0.0 ± 0.0	0.0 ± 0.0
**4**	100.0 ± 3.1	97.6 ± 4.0	92.7 ± 6.9	88.0 ± 5.6	72.3 ± 6.7	32.8 ± 6.1	5.6 ± 2.2	1.0 ± 0.4	0.1 ± 0.1
**5**	100.0 ± 4.5	96.2 ± 5.1	82.7 ± 8.4	60.3 ± 5.5	41.0 ± 6.2	24.2 ± 5.0	8.7 ± 1.6	1.2 ± 0.5	0.2 ± 0.1
**6**	100.0 ± 4.6	46.3 ± 7.4	6.8 ± 2.6	1.6 ± 0.7	1.3 ± 0.5	0.1 ± 0.1	0.3 ± 0.2	0.2 ± 0.2	0.0 ± 0.0
**7**	100.0 ± 5.3	35.1 ± 5.6	3.8 ± 1.0	0.7 ± 0.3	0.3 ± 0.1	0.1 ± 0.1	0.0 ± 0.0	0.0 ± 0.0	0.0 ± 0.0
**8**	100.0 ± 5.2	74.8 ± 6.9	34.4 ± 5.1	12.8 ± 3.0	3.4 ± 1.0	0.3 ± 0.2	0.0 ± 0.0	0.0 ± 0.0	0.0 ± 0.0
**9**	100.0 ± 3.7	62.8 ± 6.7	7.2 ± 1.9	2.0 ± 0.6	0.5 ± 0.3	0.4 ± 0.3	0.3 ± 0.2	0.0 ± 0.0	0.0 ± 0.0
**10**	100.0 ± 2.8	50.2 ± 5.1	11.0 ± 2.7	5.8 ± 2.0	2.3 ± 0.7	0.4 ± 0.3	0.2 ± 0.1	0.0 ± 0.0	0.1 ± 0.1
**11**	100.0 ± 4.0	75.2 ± 4.3	27.1 ± 3.4	12.8 ± 1.8	3.8 ± 1.0	0.2 ± 0.1	0.0 ± 0.0	0.0 ± 0.0	0.0 ± 0.0

Data was standardized and expressed as mean ± SEM (%) of 3 independent experiments with 4 parallel wells per experiment.
